# LncRNA NKILA integrates RXFP1/AKT and NF‐κB signalling to regulate osteogenesis of mesenchymal stem cells

**DOI:** 10.1111/jcmm.14759

**Published:** 2019-10-28

**Authors:** Ying Zhang, Xiangyang Cao, Peifeng Li, Yanan Fan, Leilei Zhang, Xianghao Ma, Ruibo Sun, Youwen Liu, Wuyin Li

**Affiliations:** ^1^ Medical Center of Hip Luoyang Orthopedic‐Traumatological Hospital Orthopedics Hospital of Henan Province Luoyang China

**Keywords:** AKT, NF‐κB, NKILA, osteogenic differentiation, RXFP1

## Abstract

Mesenchymal stem cells (MSCs) are previously found to have potential capacity to differentiate into osteocytes when exposed to specific stimuli. However, the detailed molecular mechanism during this progress remains largely unknown. In the current study, we characterized the lncRNA NKILA as a crucial positive regulator for osteogenesis of MSCs. NKILA attenuation significantly inhibits the calcium deposition and alkaline phosphatase activity of MSCs. More interestingly, we defined that NKILA is functionally involved in the regulation of RXFP1/PI3K‐AKT and NF‐κB signalling. Knockdown of NKILA dramatically down‐regulates the expression of RXFP1 and then reduces the activity of AKT, a downstream regulator of RXFP1 signalling which is widely accepted as an activator of osteogenesis. Moreover, we identify NF‐κB as another critical regulator implicated in NKILA‐mediated osteogenic differentiation. Inhibition of NF‐κB can induce the expression of RUNX2, a master transcription factor of osteogenesis, in a HDAC2‐mediated deacetylation manner. Thus, this study illustrates the regulatory function of NKILA in osteogenesis through distinct signalling pathways, therefore providing a new insight into searching for new molecular targets for bone tissue repair and regeneration.

**Highlights**

- NKILA functions as a crucial positive regulator of osteogenesis.
- NKILA regulates osteogenic differentiation through NF‐κB signalling‐mediated RUNX2 expression.
- RXFP1/AKT signalling is functionally involved in NKILA‐mediated osteogenic differentiation.

## 1 INTRODUCTION

Mesenchymal stem cells (MSCs), a type of pluripotent stem cells with potential ability to differentiate into multilineage cells such as osteocytes, adipocytes and chondrocyte when exposed to in vitro specific induction,^1^, ^2^, ^3^, ^4^, ^5^, ^6^, ^7^ can be isolated from multiple tissues. Due to its multipotency, MSCs‐based therapy is widely accepted as a promising candidate for clinical treatment in the area of regenerative medicine, especially bone tissue repair and regeneration. Up to date, a range of studies revealed that multiple different tissue‐derived MSCs have critical regulatory functions in the progression of bone formation, using a in vivo osteogenic animal model system.^8^, ^9^, ^10^, ^11^

Relaxin (Rln), a member of the insulin/Rln family, is functionally involved in various mechanisms associated with collagen turnover, angiogenesis and tumour metastasis.^12^, ^13^, ^14^, ^15^, ^16^, ^17^ Rln exerts autocrine, endocrine and paracrine effects through its specific membrane receptors which are detected in some reproductive tissues, as well as the bone tissues including osteoblasts, osteoclasts and osteocytes.^18^, ^19^, ^20^ Rln receptors are composed of four members (RXFP 1‐4), among which RXFP1 is previously reported to be implicated in the osteogenic differentiation of peripheral blood monocyte cells and the survival and activation of osteoclasts.^18^ Moreover, RXFP2 is found to be associated with osteoporosis.^20^ RXFP2 can bind to insulin‐like factor 3 and then augment osteoblast proliferation. These studies suggest the functional importance of Rln/RXFP signalling in the regulation of osteogenesis.^20^ AKT, a downstream regulator of Rln/RXFP, plays a critical role in osteogenesis. Prior studies reveal that the activation of AKT signalling can sustain the osteogenic differentiation of human dental follicle cells.^21^ Another critical signalling pathway involved in the regulation of osteogenesis is NF‐κB. NF‐κB, composed of homo‐ or hetero‐dimers of RelA (p65), RelB, c‐Rel, p50/p105 (NF‐κB1) or p52/p100(NF‐κB2), is previously identified as a crucial repressor for osteogenesis. Inhibition of NF‐κB dramatically enhances calcium deposition and alkaline phosphatase activity.^22^, ^23^, ^24^ Therefore, investigation on the regulation of NF‐κB activity is a key point for exploring the new strategy in clinical treatment of multiple bone tissue dysfunctions.

In this study, we first characterized the lncRNA NKILA as an important positive regulator for osteogenic differentiation using a systematic in vitro study. Loss of NKILA function significantly restrains osteogenesis of menstrual blood‐derived mesenchymal stem cells (MenSCs) and umbilical cord mesenchymal stem cells (UCMSCs). Moreover, with mRNA‐Seq screening approach, we obtain the evidence that NKILA has a regulatory role for the activation of Rln/RXFP signalling. More importantly, we demonstrated the underlying molecular basis by which NKILA regulates osteogenesis through NF‐κB pathway. This study illustrated two distinct signalling pathways by which NKILA regulates osteogenic differentiation.

## 2 MATERIAL AND METHODS

### 2.1 Isolation and culture of MenSCs and UCMSCs

The menstrual bloods were collected from the healthy female donors. Equal volume of PBS supplied with 0.25 mg/mL amphotericin B, 100 U/mL penicillin, 100 mg/mL streptomycin and 2 mM ethylenediaminetetraacetic acid (EDTA) was added to the menstrual bloods, and then standard Ficoll procedure was performed to obtain MenSCs. Subsequently, the detailed steps for MenSCs isolation and culture were conducted as previously described.^25^ For UCMSCs isolation and culture, the umbilical cord vessels were first subjected to disinfection in 75% ethanol for 1 minute and then cut it into cubes. Discard the supernatant, rinse the precipitates with DMEM and then centrifuge at 250 *g* for 5 minutes. The detailed protocol for UCMSCs isolation and culture was performed as previously reported.^25^

### 2.2 Antibodies and reagents

Anti‐IκBα (#10268‐1‐AP), anti‐HDAC2 (#12922‐3‐AP) and anti‐HDAC3 (#10255‐1‐AP) antibodies were purchased from Proteintech Group Inc, and anti‐AKT (#4685) and anti‐phospho‐AKT (#4060), anti‐GAPDH (#5174), anti‐RUNX2 (#12556) and H3K27ac (#8173) antibodies were obtained from Cell Signaling Technology (Beverly, MA, USA). The chemical reagents Bay 11‐7082 (#B5556), LY294002 (L9908), Alizarin Red S (#A5533), BCIP/NBT liquid substrate (#B1911) and the commercial osteogenic medium (#SCM121) were all from Sigma.

### 2.3 Alizarin Red S staining and ALP activity detection

For Alizarin Red staining, MenSCs were first fixed in 70% ethanol, followed by 1% Alizarin Red solution staining for 1 minute. The detailed protocol was performed as previously described.^25^ For the detection of ALP activity, cells were first fixed with 70% ethanol for 30 minutes and then subjected to the BCIP/NBT liquid substrate (0.1 mol/L 2‐amino‐2‐methyl‐1‐propanol, 1 mmol/L MgCl2 and 8 mmol/L P‐nitrophenyl phosphate disodium) incubation at 37°C for 30 minutes. The detailed procedures were carried out as previously described.^25^

### 2.4 Constructs and lentiviral infection

The shRNA targeting human NKILA were cloned into a modified pLV‐H1‐Puro lentiviral vector. The sequence for shNKILA is 5′‐ GGGCAGTAGGAAAGGAGAA‐3′. The overexpression vector of NKILA was amplified by reverse transcription PCR and then inserted into a modified pLV‐EF1α lentiviral vector as previously reported.^26^ For lentivirus infection, the detailed protocol was conducted as previously described.^26^

### 2.5 Quantitative RT‐PCR

Total RNAs were extracted from cells using Trizol reagent, followed by reverse transcription, according to manufacturers' instructions. Real‐time quantitative PCR was performed with a Master Mix kit purchased from Promega Corporation. The relative changes of gene expression were determined by the 2^−ΔΔCT^ method. The primer sequences for qRT‐PCR are as follows: F. 5′‐GGACGAGGCAAGAGTTTCAC‐3′, R. 5′‐GAGGCGGTCAGAGAACAAAC‐3′ (RUNX2); F. 5′‐CACAGCTCTTCTGACT GTCTG‐3′, R. 5′‐CTGGTGAAATGCCTGCATGGAT‐3′ (SP7); F. 5′‐AGCCAAT GATGAGAGCAATG‐3′, R. 5′‐TCCTTACTTTTGGGGTCTAC‐3′ (SPP1); F. 5′‐CATGAGAAGTATGACAACAGCCT‐3′, R. 5′‐AGTCCTTCCACGATACCAAAGT‐3′ (GAPDH); F. 5′‐GGATGAATTGGATTTAGGAA‐3′, R. 5′‐CCAAGAG GTTATGGTACA‐3′ (RXFP1); and F. 5′‐AACCAAACCTACCCACAACG‐3′, R. 5′‐ ACCACTAAGTC AATCCCAGGTG‐3′ (NKILA).

### 2.6 High throughput mRNA sequencing

The mRNA‐Seq experiments were conducted by Annoroad (Beijing, China). Total RNAs were extracted using Trizol reagent and then subjected to library construction which is prepared according to standard Illumina protocols. The libraries were sequenced with Illumina HigSeq × Ten sequence platform using the paired‐end RNA‐seq approach. For subsequent data analysis, the detailed method was performed as previously reported.^27^ The raw data have been deposited in the Sequence Read Archive (SRA) database with an accession number SRP194432.

### 2.7 Chromatin immunoprecipitation (ChIP)

Briefly, 10^7^ MenSCs were cross‐linked with 1% formaldehyde and then quenched with 125 mmol/L glycine solution. The cells were lysed, and the DNAs were sonicated into fragments from 100 to 500 bp. In the following, the sonicated lysates were cleared with high speed centrifuge, followed by co‐incubation with indicated antibodies for immunoprecipitation. Reverse the crosslinks and elute the DNAs with an elution buffer for subsequent quantification. The primer sequence of RUNX2 for ChIP‐qPCR is F. 5′‐ACCATGGTGGAGATCATCG‐3′, R. 5′‐GGCAGGGTCTTGTTGCAG‐3′.

### 2.8 Statistical analysis

All data are obtained from at least three independent experiments and shown as mean ± SD All statistical analyses were performed with Prism5 (GraphPad). Student's *t* test was used for comparisons between two groups, and one‐way ANOVA followed by Tukey *post hoc* test was used to compare more than three groups. *P*‐value < .05 was considered statistically significant.

## 3 RESULTS

### 3.1 NKILA expression is dramatically up‐regulated in response to osteogenic induction

**Figure 1:**
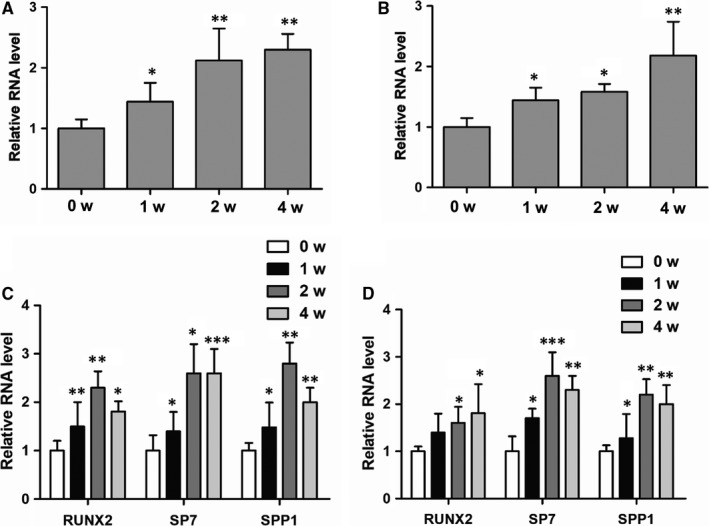
NKILA expression was elevated after osteogenic induction. A, B, Dynamic changes of NKILA expression in both MenSCs and UCMSCs were examined by qRT‐PCR assay after a commercial osteogenic medium induction for 0, 1, 2 and 4 wk. C, D, Expressions of the osteogenic marker genes in MenSCs and UCMSCs were examined, respectively, by using qRT‐PCR assay. All results are from biological triplicates and data shown are the mean ± SD. n = 3. ^*^ *P* < .05, ^**^ *P* < .01 and ^***^ *P* < .001 vs 0 w

LncRNA NKILA was previously reported to play critical roles in multiple biological processes. However, the regulatory function of NKILA in osteogenesis is still unknown. To examine the potential role of NKILA in this process, we first isolated menstrual blood‐derived mesenchymal stem cells (MenSCs) and umbilical cord mesenchymal stem cells (UCMSCs) and then performed osteogenic induction, using an osteogenic induction medium from Sigma, to observe the dynamic changes of NKILA expression. Interestingly, osteogenic induction significantly up‐regulated expression of NKILA in both of the two cell lines (Figure 1A,B), suggesting the functional importance of NKILA in the regulation of osteogenesis. In parallel, we determined expressions of osteogenesis‐associated marker genes after osteogenic induction. As expected, the representative osteogenic markers, including RUNX2, SP7 and SPP1, were markedly increased when exposed to osteogenic medium treatment (Figure 1C,D).

### 3.2 NKILA positively regulates osteogenesis of mesenchymal stem cells

**Figure 2:**
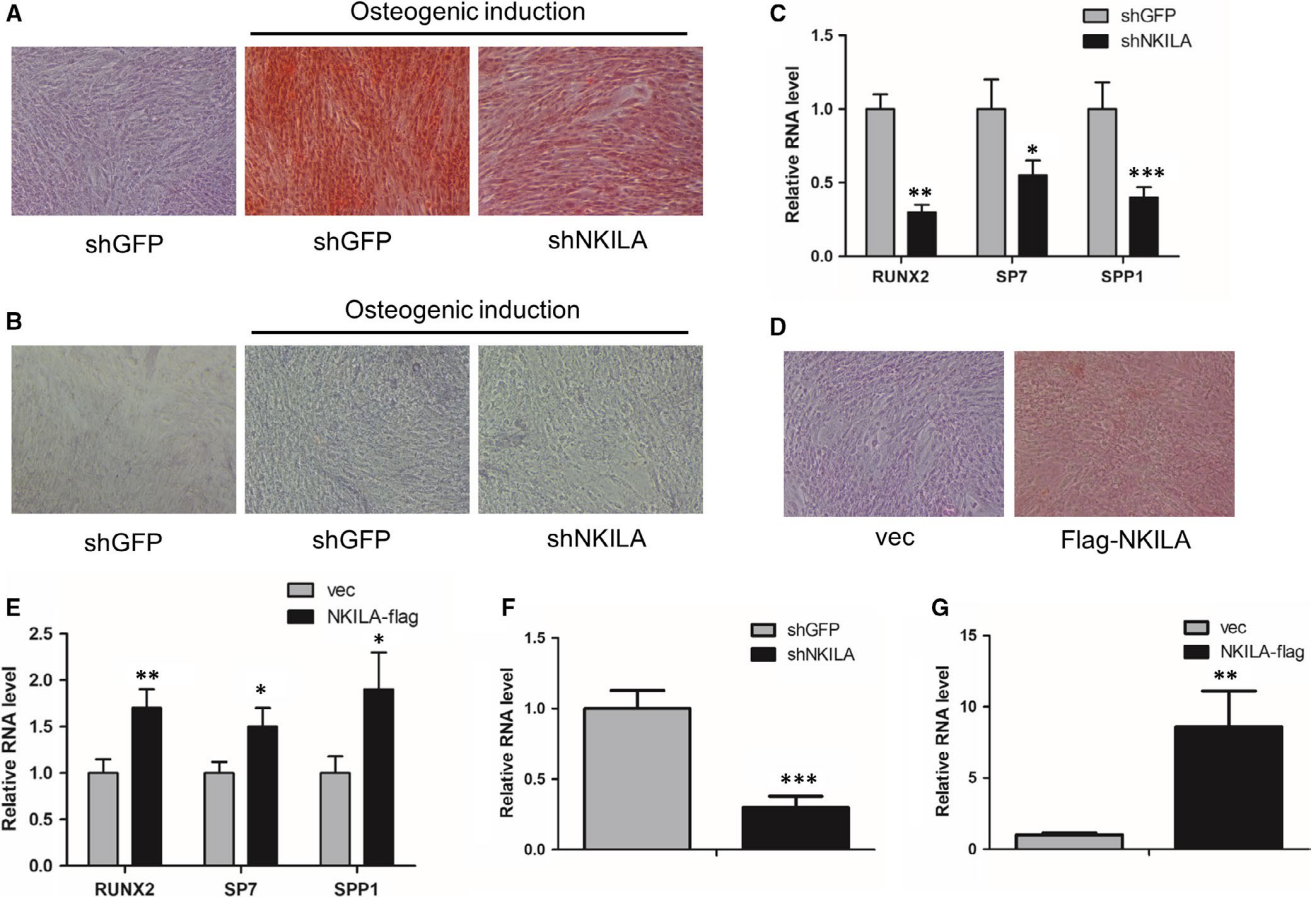
NKILA regulates osteogenesis of mesenchymal stem cells. A, The effect of NKILA on calcium deposition of MenSCs with osteogenic induction for 3 wk, determined by Alizarin Red S staining analysis after NKILA shRNA‐mediated knockdown. B, The activity of alkaline phosphatase in MenSCs, with or without NKILA knockdown, was measured by a BCIP/NBT liquid substrate system after osteogenic induction for 3 wk. C, Expressions of osteogenic markers RUNX2, SP7 and SPP1 in control and NKILA‐depleted MenSCs were examined by qRT‐PCR, respectively, after osteogenic induction for 4 d. D, The influence of overexpressed NKILA on calcium deposition of UCMSCs, assayed by Alizarin Red S staining. E, The effect of the overexpressed NKILA on representative osteogenic marker gene expressions in UCMSCs, determined by qRT‐PCR assay. F, Knockdown efficiency of NKILA in MenSCs was measured by qRT‐PCR assay. G, Overexpression efficiency of NKILA in MenSCs was examined by qRT‐PCR assay. All results are from biological triplicates and data shown are the mean ± SD. n = 3. ^*^ *P* < .05, ^**^ *P* < .01 and ^***^ *P* < .001 vs shGFP or vec

To study the effect of NKILA on osteogenesis of mesenchymal stem cells, we first generated a shRNA construct against NKILA and then performed shRNA‐mediated NKILA knockdown in both MenSCs and UCMSCs, to investigate the influence of NKILA on calcium deposition. As a consequence, Alizarin Red S staining analysis revealed that inhibition of NKILA remarkably repressed the calcium deposition of the MenSCs (Figure 2A). To further confirm the critical function of NKILA in the regulation of osteogenesis of mesenchymal stem cells, we assayed the activity of alkaline phosphatase (ALP) in MenSCs, with or without NKILA depletion. Consequently, ALP activity was significantly inhibited when NKILA was depleted (Figure 2B). Next, we determined expressions of the osteogenic markers RUNX2, SP7 and SPP1. qRT‐PCR analysis showed that attenuation of NKILA decreased expressions of these representative osteogenic marker genes (Figure 2C). Moreover, we constructed a lentivirus‐based NKILA vector and performed aberrant NKILA overexpression to observe the effect of NKILA on osteogenesis. Consistently, highly expressed NKILA obviously induced the calcium deposition of UCMSCs (Figure 2D). In addition, expressions of osteogenic marker genes were enhanced after NKILA overexpression (Figure 2E). In summary, these findings suggest that NKILA positively regulates osteogenesis of mesenchymal stem cells.

### 3.3 NKILA regulates the activity of Relaxin/RXFP1/AKT signalling pathway

**Figure 3:**
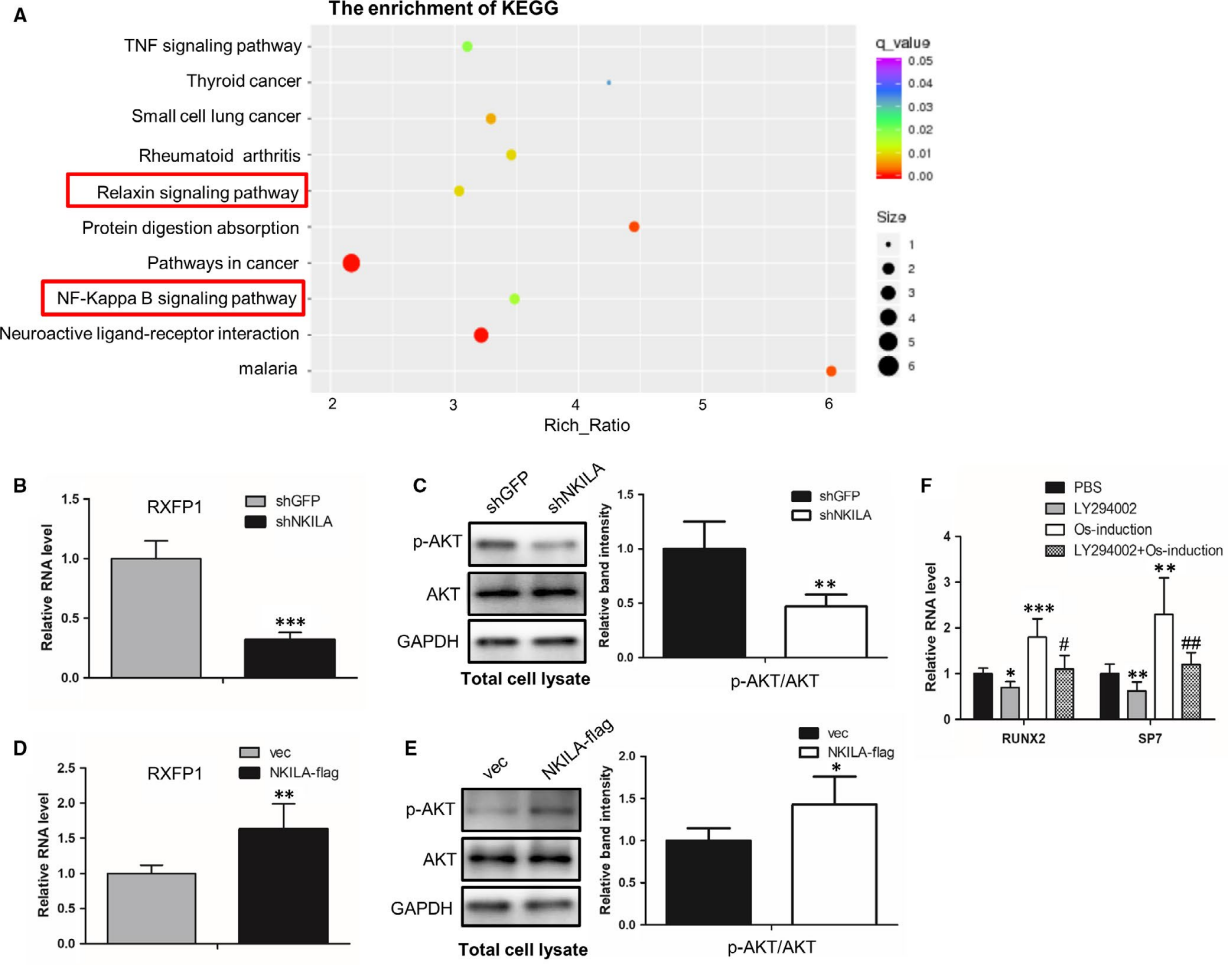
NKILA is involved in the regulation of RXFP1/PI3K‐AKT signalling. A, KEGG analysis showing the effect of NKILA on its downstream‐regulated pathways. Total RNAs were isolated from MenSCs, with or without NKILA knockdown, and then subjected to high throughput mRNA sequencing. B, The effect of NKILA on RXFP1 expression, examined by qRT‐PCR assay after NKILA knockdown in MenSCs. C, Western blot assay showing the effect of NKILA on AKT phosphorylation status. AKT activities were examined in a control and NKILA‐depleted MenSCs samples, respectively, with an antibody against phosphorylated AKT at S473 site. D, NKILA was overexpressed in UCMSCs to analyse the effect of NKILA on RXFP1 expression, using qRT‐PCR assay. E, Western blot analysis showing the effect of NKILA on the activity of AKT pathway. Enforced overexpression of NKILA was performed in UCMSCs, followed by subsequent measurement of AKT phosphorylation level at S473 site. F, The effect of AKT specific inhibitor LY294002 (10 mmol/L, 4 d) on expressions of osteogenic marker genes (RUNX2 and SP7) in UCMSCs, examined by qRT‐PCR under basal and osteogenic induction conditions for 4 d. All results are from biological triplicates and data shown are the mean ± SD. n = 3. ^*^ *P* < .05, ^**^ *P* < .01, and ^***^ *P* < .001 vs shGFP or vec or PBS; ^#^ *P* < .05 and ^##^ *P* < .01 vs osteogenic medium‐treated MenSCs

Given the crucial role of NKILA in regulation of osteogenesis, we next sought to illustrate the molecular mechanism by which NKILA regulates osteogenesis. First, we established expression profiles of MenSCs, with or without NKILA knockdown, to observe the NKILA downstream‐regulated genes and implicated signalling network. The mRNA‐Seq data analysis showed that 353 and 686 genes were up‐ and down‐regulated, respectively, after NKILA knockdown. Consistent with the above data Figure 2C, NKILA knockdown repressed expressions of the osteogenic marker genes, like RUNX2 and SPP1 (data not shown). More intriguingly, KEGG pathway analysis presented that NKILA is critically associated with Relaxin/RXFP1 signalling (Figure 3A), one of the most crucial signalling pathway implicated in the activation of osteogenesis.^18^ Therefore, we hypothesized that NKILA could regulate osteogenesis through Relaxin/RXFP1 signalling pathway. To prove this notion, we tested the expression of the critical regulator RXFP1 in MenSCs, with or without NKILA knockdown. We found that NKILA depletion markedly reduced RXFP1 expression, using qRT‐PCR assay (Figure 3B). Moreover, we examine the influence of NKILA on the activity of AKT, a downstream regulator of Relaxin/RXFP1 pathway in osteogenesis. Western blotting showed that attenuated NKILA significantly restrains the phosphorylation status of AKT at S473 site (Figure 3C), indicating the positive role of NKILA in modulating PI3K‐AKT activity. To confirm this result, we performed enforced NKILA overexpression in UCMSCs to further test the effect of NKILA on RXFP1/AKT signalling pathway. Consistently, overexpressed NKILA remarkably augmented both the RXFP1 expression and the phosphorylation status of AKT (Figure 3D,E). Next, we assayed the influence of AKT signalling on osteogenesis of UCMSCs. Identical to the prior reports, AKT inhibition with its specific inhibitor LY294002 significantly decreased expressions of the representative osteogenic markers (Figure 3F). Collectively, these observations suggest that NKILA positively regulates osteogenesis, at least in part, through Relaxin/RXFP1/PI3K‐AKT signalling pathway.

### 3.4 NKILA regulates RUNX2 expression through a NF‐κB‐mediated deacetylation mechanism

**Figure 4:**
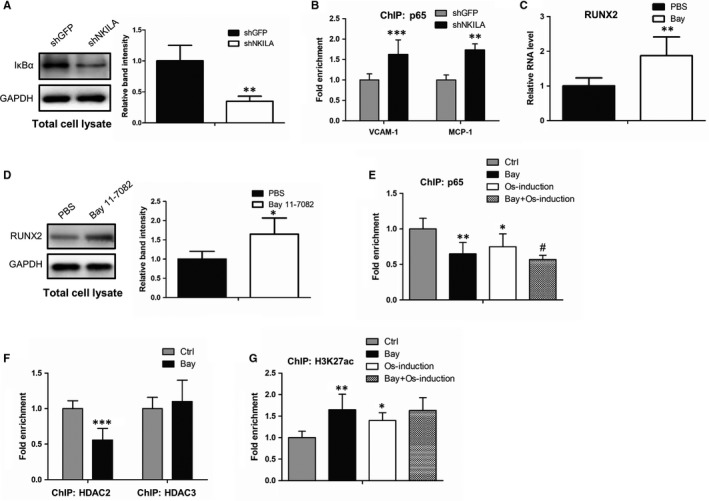
NKILA positively regulates RUNX2 expression via NF‐κB signalling. A, The effect of NKILA on IκBα expression in MenSCs, examined by western blot analysis with or without NKILA depletion. B, ChIP assays, with an antibody against p65, were performed in control and NKILA‐depleted MenSCs, respectively, to test the influence of NKILA on NF‐κB binding ability to its targeted gene promoters. C, D, The effect of NF‐κB inhibitor Bay 11‐7082 (1 μmol/L, 4 d) on RUNX2 expression in MenSCs, examined by qRT‐PCR (C) and Western blot assays (D). E, ChIP assays showing NF‐κB occupancies at RUNX2 promoter region in MenSCs with or without Bay 11‐7082 treatment (1 μmol/L, 4 d), in the presence or absence of osteogenic induction. F, ChIP assays in MenSCs showing the dynamic changes of HDAC2 and HDAC3 occupancies at RUNX2 promoter regions, respectively, upon Bay 11‐7082 treatment (1 μmol/L, 4 d). G, ChIP assays in MenSCs showing the effect of Bay 11‐7082 (1 μmol/L, 4 d) on H3K27 acetylation status at RUNX2 promoter, with or without osteogenic induction for 4 d. All results are from biological triplicates and data shown are the mean ± SD. n = 3. ^*^ *P* < .05, ^**^ *P* < .01 and ^***^ *P* < .001 vs shGFP or Ctrl or PBS; ^#^ *P* < .05 and ^##^ *P* < .01 vs osteogenic medium‐treated MenSCs

As shown in Figure 3A, NKILA is functionally involved in the regulation of NF‐κB pathway, which is identical to the prior report that NKILA functions as a critical inhibitor of NF‐κB signalling.^28^ Thus, we ask whether NF‐κB is another signalling pathway implicated in NKILA‐mediated osteogenesis. To study the precise molecular basis, we first determined the effect of NKILA on the transcriptional activity of NF‐κB. Western blot and ChIP assays showed that inhibition of NKILA promotes IκBα degradation and increased NF‐κB occupancies at its canonical target gene promoter regions (Figure 4A,B), demonstrating the transcriptional activation of NF‐κB signalling after NKILA attenuation. It is documented that NF‐κB activity has significant correlation with the expression of RUNX2,^29^, ^30^ a critical master transcription factor of osteogenesis. Thus, we ask whether NF‐κB participates in mediating RUNX2 expression. To evidence this concept, we first examined the influence of NF‐κB on RUNX2 expression. qRT‐PCR and Western blot analysis revealed that expression of RUNX2 was dramatically increased when MenSCs were treated by Bay 11‐7082, an inhibitor of NF‐κB (Figure 4C,D). Next, we tested whether NF‐κB can epigenetically regulate RUNX2 expression, as a critical transcription factor. Anti‐p65 ChIP assays were performed to examine the NF‐κB binding capacity to RUNX2 promoter. As a consequence, the chromatin‐loading of NF‐κB at RUNX2 promoter was remarkably abrogated after NF‐κB inhibitor Bay 11‐7082 treatment, both in basal and osteogenic induction conditions (Figure 4E), indicating a potential modifying role of NF‐κB to RUNX2 promoter. As is well reported, NF‐κB can help recruit specific histone deacetylase, such as HDAC2 and HDAC3, therefore facilitating deacetylation of histones and co‐repressing gene transcription.^31^, ^32^ To test this possibility, we performed anti‐HDAC2 and HDAC3 ChIP assays and found that the occupancy of HDAC2, but not HDAC3, at RUNX2 promoter was markedly restrained after NF‐κB inhibition (Figure 4F). Furthermore, we assayed the influence of NF‐κB on acetylation status of histone 3 at K27 site (H3K27), a key modification site responsible for gene transcriptional activation. Identically, MenSCs with Bay 11‐7082 treatment revealed an increased acetylation level of H3K27 at RUNX2 promoter (Figure 4G). Taken together, these observations demonstrate a NF‐κB‐mediated deacetylation mechanism by which NKILA positively regulates the expression of RUNX2, therefore inducing the process of osteogenic differentiation.

## 4 DISCUSSION

LncRNA NKILA is a previously documented regulator, functionally involved in multiple pathological processes, such as cancer and immunity. However, the regulatory role NKILA in osteogenesis has not been identified up to date. In this study, we first characterized NKILA as a critical inducer during osteogenic differentiation of mesenchymal stem cells (MSCs). Gain or loss of NKILA function study revealed that NKILA could positively regulates the calcium deposition and alkaline phosphatase activity of MSCs, indicating a stimulatory role of NKILA in the process of osteogenesis.

Relaxin/RXFP1 signalling is previously found to be functionally involved in the regulation of osteogenesis. The activation of Relaxin/RXFP1 can markedly induce osteogenic differentiation.^18^ By using mRNA sequencing approach, we first identified NKILA as a critical upstream regulator of Relaxin/RXFP1 signalling. NKILA is found to positively regulate the expression of RXFP1. Furthermore, we demonstrate that NKILA could dramatically enhance the activity of AKT, another crucial positive regulator of osteogenic differentiation,^21^, ^33^, ^34^ and a downstream regulator of Relaxin/RXFP1 pathway. Consistent with the prior reports,^21^, ^33^ inhibition of AKT activity obviously reduces the expressions of osteogenic marker genes, further demonstrating the functional importance of Relaxin/RXFP1/AKT signalling pathway during osteogenic differentiation. As a critical regulator of Relaxin/RXFP1/AKT signalling, we conclude that NKILA can regulate the osteogenic differentiation, at least in part, through Relaxin/RXFP1/AKT pathway.

Increasing evidences reported that NKILA can serve as a critical suppressor of NF‐κB signalling.^26^, ^28^, ^35^ The activation of NF‐κB can restrain osteogenesis.^22^, ^23^, ^24^ However, the molecular basis by which NF‐κB inhibits osteogenic differentiation is still unclear until now. In the present study, we characterize NF‐κB as an upstream regulator of RUNX2, which is a critical master transcription factor of osteogenesis. NF‐κB is found to bind to RUNX2 promoter and helped to recruit the histone deacetylase HDAC2, therefore epigenetically restraining the acetylation level of histone 3 at the K27 site, finally leading to the transcriptional repression of RUNX2. Moreover, we further confirmed the inhibitory role of NKILA for the activity of NF‐κB through regulating the proteasome‐mediated IκBα degradation. Based on these observations, we conclude that NKILA can regulate osteogenic differentiation partially in an NF‐κB–mediated RUNX2 expression manner.

**Figure 5:**
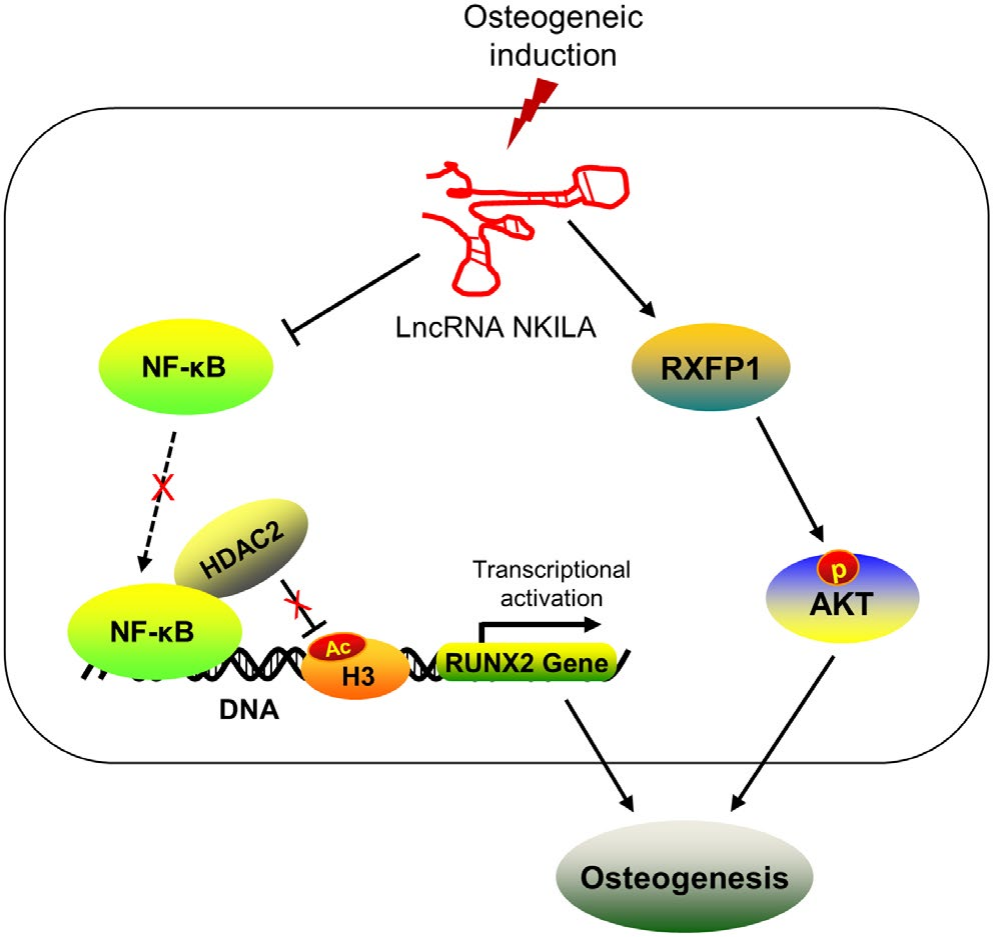
Schematic representation of the molecular mechanism by which NKILA regulates osteogenesis. During osteogenesis of MSCs, the lncRNA NKILA is induced and plays a critical positive role in the regulation of osteogenic differentiation. As a crucial repressor of NF‐κB signalling, NKILA can block the recruitment of NF‐κB to promoter of the osteogenic regulator RUNX2 and subsequently restrains NF‐κB‐mediated HDAC2 deacetylation modification at H3K27 site, epigenetically activating RUNX2 gene transcription. On the other hand, NKILA is involved in the regulation of RXFP1/AKT signalling pathway. NKILA can positively regulate RXFP1 expression, therefore elevating the activity of its downstream regulator AKT, a crucial positive signalling during osteogenesis. Overall, NKILA promotes osteogenic differentiation, at least in part, via NF‐κB and RXFP1/AKT signalling

In summary, this study identifies two critical signalling pathways, through which NKILA facilitates the osteogenesis of MSCs (Figure 5), providing a new insight into understanding the regulatory function of NKILA during osteogenic differentiation. More importantly, this study may offer us a promising molecular target for the clinical treatment of bone tissue repair and regeneration.

## CONFLICT OF INTEREST

The authors declare that there is no conflict of interest.

## AUTHOR CONTRIBUTIONS

Y. Z. and W. L. conceived and designed the project. L. Z., X. M. and R. S. contributed to acquisition of data. Y. Z. and Y. L. helped in data analysis. X. C. and P. L. performed statistical analysis. P. L. and Y. F. wrote the draft of the manuscript. Y. F. and X. C. helped revising the manuscript. All authors contributed to discussions.

## ETHICS APPROVAL AND CONSENT TO PARTICIPATE

All the MenSCs and UCMSCs were obtained with the informed consent of the donors. All experiments in this manuscript meet the “Declaration of Helsinki” and were approved by Ethics Committee of the Orthopedics Hospital of Henan Province.

## Data Availability

The data are available from the corresponding author upon reasonable request.
